# Optimizing SOI Slot Waveguide Fabrication Tolerances and Strip-Slot Coupling for Very Efficient Optical Sensing

**DOI:** 10.3390/s120302436

**Published:** 2012-02-23

**Authors:** Vittorio M. N. Passaro, Mario La Notte

**Affiliations:** Photonics Research Group, Dipartimento di Elettrotecnica ed Elettronica, Politecnico di Bari, Via Edoardo Orabona 4, 70125 Bari, Italy; E-Mail: lanottemario@alice.it

**Keywords:** slot waveguides, homogeneous optical sensing, slot couplers, 07.07.Df Sensors, 42.79.Gn Optical waveguides and couplers

## Abstract

Slot waveguides are becoming more and more attractive optical components, especially for chemical and bio-chemical sensing. In this paper an accurate analysis of slot waveguide fabrication tolerances is carried out, in order to find optimum design criteria for either homogeneous or absorption sensing mechanisms, in cases of low and high aspect ratio slot waveguides. In particular, we have focused on Silicon On Insulator (SOI) technology, representing the most popular technology for this kind of devices, simultaneously achieving high integration capabilities, small dimensions and low cost. An accurate analysis of single mode behavior for high aspect ratio slot waveguide has been also performed, in order to provide geometric limits for waveguide design purposes. Finally, the problem of coupling into a slot waveguide is addressed and a very compact and efficient slot coupler is proposed, whose geometry has been optimized to give a strip-slot-strip coupling efficiency close to 100%.

## 1. Introduction

Nowadays, rapid advancements in photonic technologies have significantly enhanced the performance of photonic biochemical sensors, particularly in the areas of light-analyte interaction, device miniaturization, multiple analysis and integration. Due to these reasons, optical biosensors are becoming essential in crucial areas of applications such as environmental monitoring, biotechnology, medical diagnostics, security, drug screening and food safety, to name but a few. Moreover, the more and more increasing demands for low cost, reliable and multi-function sensors has brought an extensive industrial and scientific interest in photonic sensors due to their high integration. To this purpose, an accurate choice of materials and device architectures assumes a very important role. High refractive index (HI) materials, such as silicon and other group IV materials, are very promising for integrated optical sensors for their capability to provide very high light confinement, low propagation losses and bend losses reduction in ring resonator-based sensors. On the other hand, low refractive index (LI) materials, such as water or other liquid substances, can be very useful for sensing purposes. For example, a number of chemical and biochemical species can be easily dissolved in the aqueous solution involving a change of the solution refractive index, directly related to the analyte concentration. In recent years, slot waveguides have attracted a lot of interest for their capability to combine the advantages of both HI and LI materials, resulting in a significant performance improvement compared to sensors based on standard photonic wire waveguides [1–11]. Due to the nanometer dimension of slot waveguides, an accurate analysis of fabrication tolerances becomes critical in order to achieve a robust and optimized sensor design. By this way, we have performed an accurate sensitivity analysis, taking into account the influence of a number of technological parameters. Another critical aspect in sensor design is the strip-slot waveguide coupling [12–14]. In fact, light cannot be injected directly into the slot waveguide but it needs to be launched in an input photonic strip, which carries light to a properly designed strip-slot waveguide coupler. The mode conversion between photonic strip and slot waveguide must be adiabatic in order to avoid any undesired back-reflection, responsible of sensor performance decrease. A very compact and efficient solution for strip-slot waveguide coupling is presented, particularly suitable for Lab-On-a-Chip applications in Silicon-On-Insulator (SOI) technology.

## 2. Sensing Mechanisms

A wide variety of chemical species (analytes), dissolved in a proper solution or solvent, can be detected by taking advantage of the induced refractive index change of the solvent, which depends on the analyte concentration. This sensing mechanism is known as homogeneous sensing. In optical sensors the solution usually covers the whole photonic structure, acting as a cladding medium for the waveguide. Due to this reason, an analyte concentration variation can induce an effective refractive index variation of the waveguide propagating mode. According with variational theorem for dielectric waveguides, sensitivity in homogeneous sensing can be written as [9]:

(1) $$S={\frac{{\partial n}_{\mathrm{eff}}}{{\partial n}_{c}}|}_{n_{c}=n_{c}^{0}}=\frac{{2n}_{c}^{0}}{Z_{0}P}\iint_{C}{\left|\vec{E}(x,y)\right|}^{2}\;\mathrm{dxdy}=\frac{{2n}_{c}^{0}\iint_{\infty }{\left|\vec{E}(x,y)\right|}^{2}\mathrm{dxdy}}{Z_{0}P}\Gamma_{c}^{I}$$

where:

(2) $$P=\iint_{\infty }\left[\left(\vec{E}\times {\vec{H}}^{*}+{\vec{E}}^{*}\times \vec{H}\right)\cdot \hat{z}\right]\mathrm{dxdy}$$

and *Z_0_* is the free space impedance, *n_eff_* is the effective mode index, *n_c_* is the solution refractive index, *n_c_^0^* is the solution refractive index in absence of the analyte, Γ_c_^I^ is the optical field intensity confinement factor in cladding region, *Ē* and *H̄* are the electric and magnetic field vector, respectively. The integration domains C and ∞ stands for cladding cross section and whole computational region, respectively.

Equation (1) shows a direct proportionality between sensitivity and intensity confinement factor in the cladding region. Another important consideration is related to the choice of the cladding material. It is evident from Equation (1) that sensitivity is directly proportional to the value of *n_c_^0^*, so it could appear that a higher value of the cladding refractive index would make the sensitivity larger. On the other hand, it is important to remember that the lower the cladding refractive index, the higher the electric field amplitude at the inner boundaries of the gap regions, the higher the confinement factor, so a proper choice between conflicting requirements has to be performed. For chemical sensing operating in near infrared range, an aqueous solution as cladding medium is a common situation in many applications.

Another very promising sensing scheme concerns the absorption principle. In fact, many dangerous gases or volatile organic contaminants, either in air or in aqueous solution, can be detected with ultra-high selectivity and immunity to electromagnetic interferences by optical absorption spectroscopy [15,16]. Such a sensing principle exploits the fact that each contaminant has very well defined vibrational absorption bands, which can be considered as fingerprints of the particular compound.

Slot waveguides are very promising devices for absorptive sensing purpose, since they are able to confine a very high field power percentage in the cladding medium. By this way, the optimization of the power confinement factor into the cladding medium becomes crucial.

It is very important to observe that, in general, optical field power (Γ_c_ in cladding region) and intensity (Γ_c_^I^ in cladding region) confinement factors are different and can show different variations with respect to the cladding refractive index, due to the non-zero z-component of the electric field. Power and intensity confinement factors can be defined as:

(3) $$\Gamma_{c}=\frac{\text{Re}\left\{\iint_{C}\left(\vec{E}\times {\vec{H}}^{*}\right)\cdot \hat{z}\;\mathrm{dxdy}\right\}}{\text{Re}\left\{\iint_{\infty }\left(\vec{E}\times {\vec{H}}^{*}\right)\cdot \hat{z}\;\mathrm{dxdy}\right\}}\;;\;\Gamma_{c}^{I}=\frac{\iint_{C}{\left|\vec{E}(x,y)\right|}^{2}\mathrm{dxdy}}{\iint_{\infty }{\left|\vec{E}(x,y)\right|}^{2}\mathrm{dxdy}}$$

## 3. Slot Waveguides for Sensing: Fabrication Tolerances

**Figure 1.**
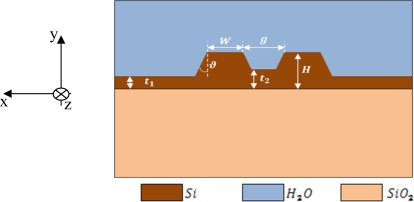
Schematic view of slot waveguide cross section. *H* and *W* are photonic wires height and width, respectively, *g* is the gap width, *t_1_* and *t_2_* are silicon etching residues outside and inside the gap region, respectively, and *ϑ* is the slanted sidewall angle.

A slot waveguide is a well known optical waveguide, formed by two high refractive index photonic wires very close to each other, surrounded by a low refractive index medium. Our analysis is based on Silicon-On-Insulator (SOI) technology, so we assume buried oxide substrate (*n_s_* = 1.444), silicon photonic wires (*n_w_* = 3.476) and an aqueous solution as cladding (*n_c_* = 1.330). The operative wavelength is λ = 1.55 μm. The entire structure is sketched in Figure 1.

The design of an efficient slot waveguide sensor [4,5,7,9] is strictly connected to the slot waveguide design and its fabrication tolerance. In this section we present some remarkable considerations for an optimum waveguide design in SOI technology. The fabrication tolerances are taken into account defining the technological parameter Ψ_j_ as in Equation (4):

(4) $$\Psi_{j}={\frac{\partial X}{\partial t_{j}}|}_{t_{j}=t_{j}^{0};t_{i}=0\forall i\neq j}$$

where *X* can be either Γ_c_ or *S*, depending on the sensing mechanism (*i.e.*, absorption or homogeneous, respectively), *t_j_* is the dimension of the *j*th parameter and $t_{j}^{0}$ is a reference value, *i.e.*, the value assumed in the designed ideal structure for that parameter.

Such a definition has been proved to work very well for several technological parameters. However, both Γ_c_ and *S* do not exhibit a linear dependence on slanted sidewall angle (Ψ_ϑ_ parameter), if a large angular range (between 0° and 10°) is considered. A more efficient definition can be found to be the slope of the line connecting the sensitivity values corresponding to *ϑ* = 0 and *ϑ* = *ϑ_max_*, being *ϑ_max_* the maximum angle considered in the optimization process. Unless otherwise specified, in our analysis we consider *ϑ_max_* = 10°, according to SOI technology state-of-the-art [9,17,18]. In next paragraphs it will be evident how this is a worst case approximation, since it always overestimates the sensitivity decrease in the analyzed angular range. Nevertheless, this definition of Ψ_ϑ_ enables to predict well the influence of optimizing parameters in terms of fabrication tolerances.

When fabricating a slot waveguide, many parameters need to be optimized. In this work, both power confinement factor into the cladding region (Γ_c_) and sensitivity (*S*) are assumed as design figures of merit, since they are directly correlated with the performances of the photonic sensor. In fact, while sensitivity is related to the quality of the sensor for homogeneous sensing scheme, power confinement factor is the fundamental figure of merit when considering the absorption sensing scheme: the higher the value of Γ_c_, the higher the percentage of optical power interacting with the substance to be analyzed (for example, a specific liquid solution, enzyme or gas).

It can be demonstrated that sensitivity and power confinement factor are related to each other by Equation (5), being *x-y* the waveguide cross section and *z* the propagation direction (as in Figure 1).

(5) $$S≅\frac{n_{c}^{0}}{n_{\mathrm{eff}}}\left(\Gamma_{c}+\frac{\iint_{C}{\left|E_{z}\;(x,y)\right|}^{2}\mathrm{dxdy}}{\iint_{\infty }\left({\left|E_{x}\;(x,y)\right|}^{2}+{\left|E_{y}\;(x,y)\right|}^{2}\right)\mathrm{dxdy}}\right)=\frac{n_{c}^{0}}{n_{\mathrm{eff}}}\left(\Gamma_{c}+\gamma_{z}\right)$$

Since both *n_eff_* and *γ_z_* vary depending on the slot waveguide cross section, the two figures of merit Γ_c_ and *S* lead, in general, to different criteria for optimized designs in terms of fabrication tolerances.

Furthermore, slot waveguide performances are very closely related to the height of the two silicon wires. It is clear that the higher the silicon wires, the largest the refractive index discontinuity area, the largest the sensitivity and the power confinement factor. On the other hand, when silicon wires becomes too thick, several higher order TE-like guided modes (*i.e*., slot modes) start to be supported by the structure, which could have a damaging effect for sensing purposes, for example if an interferometer scheme is used for amplitude interrogation.

We have performed an accurate analysis on both low and high aspect ratio (height/width ratio) slot waveguides, showing advantages and differences in terms sensitivity, power and intensity confinement factors and fabrication tolerances.

### 3.1. Low Aspect Ratio Slot Waveguides: Absorption Sensing

In this section, we focus on SOI slot waveguides with low aspect ratio, being the slot height *H* = 220 nm, *i.e.*, a typical value for standard SOI waveguides. In order to maximize the optical power confined into the cladding region (for *absorption sensing* schemes), an investigation of Γ_c_ as a function of photonic wires width (*W*) and gap (*g*) has been carried out by using the full vectorial 2D Finite Element Method (FEM) approach [19].

**Figure 2.**
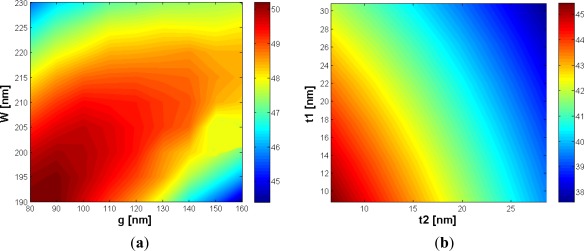
(**a**) Γ_c_ (%) *versus W* and *g* with *H* = 220 nm; (**b**) Γ_c_ (%) *versus t_2_* and *t_1_* with *H* = 220 nm, *W* = 210 nm and *g* = 120 nm.

The gap region has been varied in the range between 80 and 220 nm and the silicon wires width between 190 and 230 nm. The analysis shows that an optimum region for Γ_c_ exists, with values up to 50%, as shown in Figure 2(a). The reason of this behavior can be easily understood considering that, for larger values of *W*, the optical field is better confined into both silicon wires, so the values of the field at the boundary with the slot region is reduced and the power confinement decreases. On the other hand, if the silicon wires width becomes too small, the optical field will penetrate more into the substrate, so the cladding confinement factor will again decrease, simultaneously increasing the oxide confinement factor.

About the gap region, it is intuitive that for larger values of *g* the electromagnetic evanescent fields will weakly overlap into the slot region and the value of Γ_c_ will decrease. On the other hand, if the gap dimension becomes too small, the effective index of the slot waveguide mode increases approaching the value proper to a waveguide of width *2W*, so the silicon confinement factor increases, too.

It can be found from Figure 2(a) that, for *W* ranging from 190 nm to 230 nm and *g* from 80 nm to 160 nm, the slot waveguide can approach a cladding confinement factor between 45% and 50%. However, there are several limitations to the gap width. First of all, gap dimension less than 100 nm are difficult to be achieved in practice, due to technology limitations. Secondly, if the gap dimension becomes too small, the slot mode reaches the cut-off condition and, finally, the smaller the gap, the smaller the amount of particles interacting with the propagating light. Due to these reasons, our optimization focuses on gap values *g* > 100 nm. In such a range, an optimum choice for *W* can be found to be 210 nm, always ensuring Γ_c_ > 49% for *g* ranging between 110 and 160 nm. The maximum value of power confinement factor has been found to be 49.41% for *g* = 120 nm.

All the above discussed considerations refer to a slot waveguide without any silicon etching residue and with perfectly vertical silicon wire sidewalls. In fact, the possibility to have a silicon etching residue both inside and outside the gap region is an important aspect to be considered and a possible situation in the etching process, limiting the field confinement factor into the slot region. Then, the influence of a possible silicon residue of thickness *t_1_* outside the slot region, and *t_2_* inside the slot region (see Figure 1) has been again investigated by FEM.

The result of this analysis shows that the influence of a silicon residue inside the gap region is typically stronger than the influence of a silicon residue outside the slot region (Figure 2(b)), which is a logical consequence of the fact that the optical field is mostly concentrated in the slot region. To give an idea, a silicon layer (both inside and outside the gap region) with a thickness of 10% of silicon wire height can reduce the confinement factor of more than 10%, so particular attention needs to be paid in the etching process. However, silicon etching residues outside the gap region can be eliminated more easily than etching residue inside the slot region, so this aspect can be reasonably neglected.

**Figure 3.**
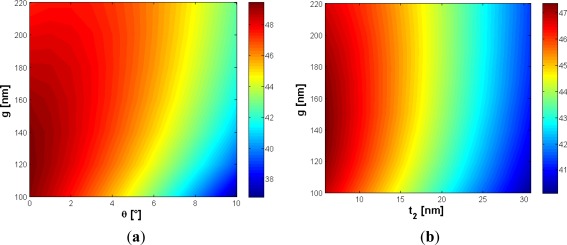
(**a**) Γ_c_ (%) *versus ϑ* and *g*, with *H* = 220 nm and *W* = 210 nm; (**b**) Γ_c_ (%) *versus t_2_* and *g* with *H* = 220 nm, *W* = 210 nm, and *t_1_* = 5 nm.

In order to reduce the effect of the etching residue (*t_2_*) on the cladding confinement factor, the influence of the gap dimension has been investigated. In fact, the larger the gap, the smaller the influence of the silicon residue, as evident in Figure 3(b). In particular, the choice of *g* = 120 nm ensures Ψ_t_2__ = −0.2648 %/nm, very close to the value obtained for *g* = 160 nm (*i.e.*, −0.2493 %/nm), so any practical advantage does not occur by increasing the gap dimension with respect to the etching residue issue. Furthermore, an accurate analysis of the effect of the slanted sidewalls has been performed. The contour map in Figure 3(a) shows the cladding confinement factor *versus* the gap dimension and the slanted walls angle *ϑ*. This angle is measured starting from the perfectly vertical sidewalls, as shown in Figure 1. Of course, the effect of the slanted sidewalls is to reduce the surface of the gap region. It could be observed, from Figure 3(a), that the influence of slanted walls is strongly reduced with increasing the gap dimension. For example, for *g* = 100 nm and *ϑ* = 8°, Γ_c_ decreases with respect to the ideal case (*ϑ* = 0°) of about 9%, while Γ_c_ is reduced by less than 5.4% with *g* = 160 nm. Accordingly, the absolute value of Ψ_ϑ_ decreases for wider gaps. In particular, a gap of 100 nm guarantees Ψ_ϑ_ = −1.24 %/°, while a wider gap *g* = 160 nm ensures Ψ_ϑ_ = −0.75 %/°. It should be pointed out that these coefficients represent a worst case estimation. Obviously, the optimal design should depend on the available technology, anyway *g* = 160 nm has been found as a good trade-off choice since the confinement factor never falls below 42% for ϑ within 10°.

**Figure 4.**
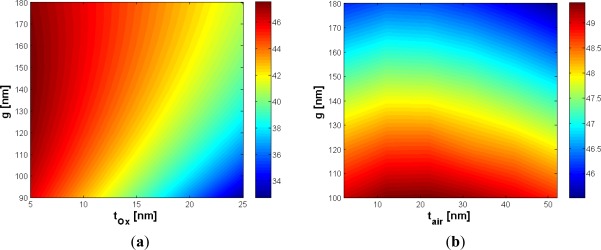
(**a**) Γ_c_ (%) *versus t_ox_* and *g*, with *H* = 220 nm and *W* = 210 nm; (**b**) Γ_c_ (%) *versus t_air_* and *g*, with *H* = 220 nm and *W* = 210 nm.

Nowadays, waveguide sidewall roughness is an important aspect to be considered for reducing the optical losses, requiring critical control of technological process. Using optimized SOI technology processes, sidewall roughness with standard deviation as low as 1.5 nm and correlation length of 13 nm can be successfully achieved [20,21]. Moreover, deposition of an additional oxide layer or thermal oxidation has been demonstrated in a number of works to mitigate the light scattering loss at the roughness [22]. Due to this reason, the effect of an oxide layer, a few nanometers thick (*t_ox_*), on the structure top has been expressly discussed and optimized in the paper. The additional oxide layer has obviously the effect to reduce the sensitivity since it fills part of the gap region, simultaneously decreasing the refractive index contrast with respect to the silicon wires. Figure 4(a) shows that the influence of the oxide layer could be mitigated by increasing the slot width. The Ψ_t_ox__ coefficient is −0.4922 %/nm, calculated for *g* = 120 nm, while a value of −0.3601 %/nm can be achieved for *g* = 160 nm.

Then, the influence of a possible air bubble, embedded into the gap slot region with a height of *t_air_*, has been also analyzed. It could be due to imperfections of the slot region and has a specific importance in case of time-dependent measurements [1], with a twofold effect on the field confinement factor. In fact, the air bubble fills part of the gap region, limiting the slot area but, at the same time, an air bubble located in the bottom of the slot region strongly reduces the field penetration into the buried oxide layer.

This effect is obviously related to the increase of the field attenuation coefficient in the *y*-direction, because the oxide layer has a higher refractive index than the air bubble. In practice, for values of *t_air_* sufficiently small compared with the slot height, the air bubble acts as a buffer layer, whose influence is to increase the cladding confinement factor, so a peak as in Figure 4(b) should be expected. This peak occurs for a bubble dimension at about 22 nm (10% of the slot height), quite regardless of the gap dimension. Furthermore, for air bubble dimension > 25 nm, the sensitivity has been found to linearly decrease with *t_air_*. In order to give a worst case estimation of sensitivity decrease, the calculated Ψ_t_air__ coefficients refer to the negative slope which occurs for t_air_ > 25 nm. For *g* = 120 nm and *g* = 160 nm, Ψ_t_air__ coefficients are −0.0231 %/nm and −0.0240 %/nm, respectively. Table 1 summarizes the most significant results of our optimization.

### 3.2. Low Aspect Ratio Slot Waveguides: Homogeneous Sensing

**Figure 5.**
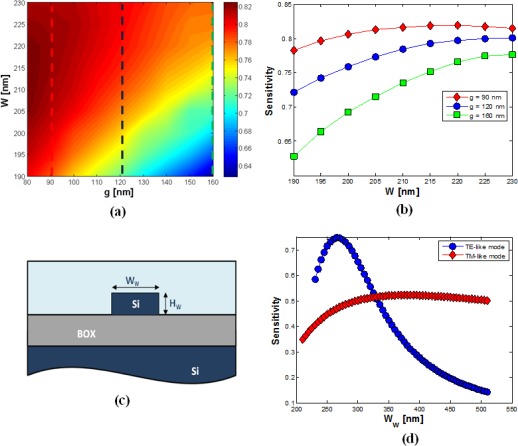
(**a**) Sensitivity *versus W* and *g* with *H* = 220 nm; (**b**) Sensitivity *versus W* for different values of gap region width; (**c**) Wire waveguide cross section; (**d**) Sensitivity of TE-like and TM-like modes *versus W_w_* for a silicon wire waveguide with *H_w_* = 220 nm.

In this paragraph, we focus our analysis on design criteria for *homogeneous sensing* optimization. Then, the sensitivity achieved by the slot waveguide, for the same range of values of *W* and *g* of Figure 2(a), has been calculated and the results have been reported in Figure 5(a,b). It is important to note that increasing values of the gap dimension tend to significantly reduce the waveguide sensitivity.

On the other hand, all discussed limitations regarding the lower limit for *g* still hold. It could be observed in Figure 5(b), that the sensitivity is always >0.72 for a gap width of 120 nm, and a maximum value of roughly 0.8 can be achieved for *W* = 220 nm. Although this choice of *g* is not optimal in terms of sensitivity, the value reached with such a design is very close to the maximum value for *S* (∼0.82) in the investigated range, simultaneously avoiding the problems related to small gap values, as discussed above. Furthermore, this gap dimension makes the sensitivity quite insensitive to any variation of *W* in the range between 210 and 240 nm, which represents a very desirable condition since such a dimension is technologically defined by the etching process. It is remarkable that the sensitivity in slot waveguides is considerably higher than that achievable in silicon wire waveguides, whose cross section is sketched in Figure 5(c). This circumstance is proved by comparing Figure 5(b,d) for *H = H_w_* = 220 nm. It should be noted that slot waveguides with *g* ≤ 160 nm always exhibit a sensitivity higher than the maximum achieved in silicon wires (∼0.74 for *W_w_* = 265 nm) for *W* ≥ 215 nm. Moreover, silicon wires exhibit their peak sensitivity for a width very close to the TE-like cut-off condition, so requiring a critical control of fabrication process. Finally, slot waveguides are much more tolerant to *W* variations. In fact, the TE-like sensitivity curve of the wire waveguide is characterized by a very fast decrease, compared to that found for slot waveguides.

**Figure 6.**
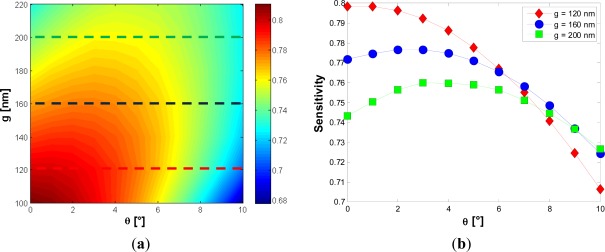
(**a**) Calculated Sensitivity *versus g* and *ϑ*, with *W* = 220 nm and *H* = 220 nm; (**b**) Sensitivity *versus ϑ* for different values of *g*.

Referring to slot waveguides, assuming a silicon wire width of 220 nm and a reference gap dimension of 120 nm, a very low sensitivity dependence with respect to *g* has been found, which guarantees high tolerance to any possible, technologically induced, variations of the gap size. It is interesting to note that this result is obtained for silicon wires having squared cross sections (220 × 220 nm^2^). As in case of confinement factor, fabrication tolerances have been calculated for the sensitivity with respect to several technological parameters. The optimizing parameter is *g*, while the silicon wires width is *W* = 220 nm. Firstly, the influence of sidewall angles on sensitivity has been investigated for different slot widths. Figure 6(a,b) shows that, with increasing the gap dimension, a peak in the sensitivity curve can be found for a specific value of *ϑ*.

A sensitivity slope increase with slightly increasing both sidewall angles and gap dimensions can be noted. The effect of sidewall angle on sensitivity is twofold. Once fixed the slot gap *g* on the top of the slot waveguide (Figure 1), it is clear that slanted sidewalls have the effect of filling part of the gap region with silicon (n_w_ = 3.476). As a result, the effective index of the slot mode tends to increase, simultaneously resulting in an increase of the field confinement factor into the silicon wires (Γ_Si_). According with this consideration, the sensitivity should decrease. On the other hand, when slanted walls are considered, the gap *g* becomes a linear function of the y coordinate, according with:

(6) $$g_{\vartheta }(y)=g+2(y-H)\;\text{tan}(\vartheta ),\;\;0\leq y\leq H$$

The average value of the gap is then reduced with respect to the value proper to a slot waveguide with vertical sidewalls, according to:

(7) $$\langle g_{\vartheta }\rangle =g-H\;\text{tan}(\vartheta )$$

**Figure 7.**
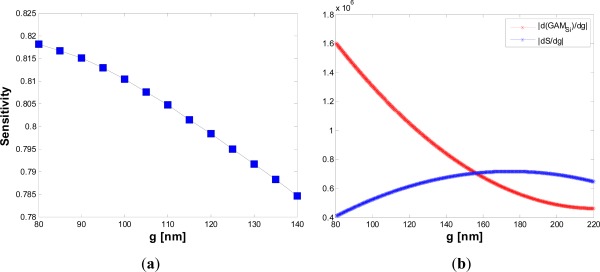
(**a**) Sensitivity *versus g* with *H = W* = 220 nm; (**b**) dS/d*g* and dΓ_Si_/d*g versus g*.

Due to this decrease, the field experiences an amplitude enhancement in the low index medium, which should lead to an increased sensitivity. Since the two effects discussed above act simultaneously, a peak in the graph in Figure 6(b) is expected. However, it is important to consider the specific influence of *g* on sensitivity to explain why the peak occurs only for wider gaps. Figure 7(a) shows the sensitivity *versus* the gap dimension for *W* = 220 nm and *H* = 220 nm.

**Figure 8.**
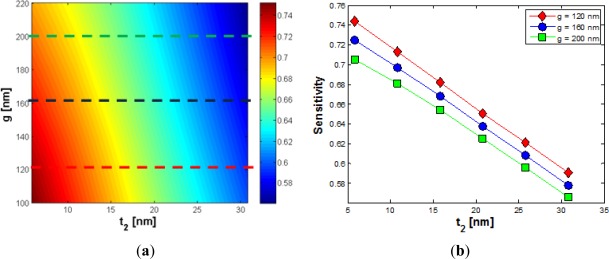
(**a**) Sensitivity *versus g* and *t_2_*, when *W* = 220 nm, *H* = 220 nm and *t_1_* = 5 nm; (**b**) Sensitivity *versus t_2_* for different values of *g*.

Figure 7(b) shows clearly that the smaller the gap region, the smaller the absolute value of the sensitivity derivative with respect to *g* (blue curve). The reason of this behavior has been discussed before. This observation explains well the different behavior of the red and green curves in Figure 6(b). In fact, for large gaps (*g* > 150 nm), the sensitivity increases significantly due to the decrease of 〈*g_ϑ_*〉, while for small gap values (*g* ∼ 120 nm) the dominant effect is an increase of the field confinement factor into the silicon wires (Figure 7(b)), so the red curve of Figure 6(b) does not exhibit any peak. We have found Ψ_ϑ_ = −0.009203 deg^−1^ for *g* = 120 nm, while a value of Ψ_ϑ_ = −0.00036 deg^−1^ can be achieved for *g* = 220 nm. In Figure 8(a,b), the influence of the silicon etching residues on sensitivity is shown.

Figure 8(b) shows how sensitivity linearly decreases with respect to *t_2_*, with a slope quite independent from the gap value. For gaps of 120 and 220 nm, Ψ_t_2__ = −0.00612 nm^−1^ and −0.00542 nm^−1^ have been calculated, respectively, so it is seen that gap dimension has a poor influence on silicon residue. In addition to *t_2_*, other critical parameters to be taken into account in the sensor design are the thickness of a possible additional oxide layer (thickness *t_ox_*), covering the whole structure top, and the dimension (*t_air_*) of a possible air bubble embedded into the gap region.

Of course, the influence of both these parameters should be to reduce the sensitivity, for the same reasons discussed above in case of power confinement factor optimization.

**Figure 9.**
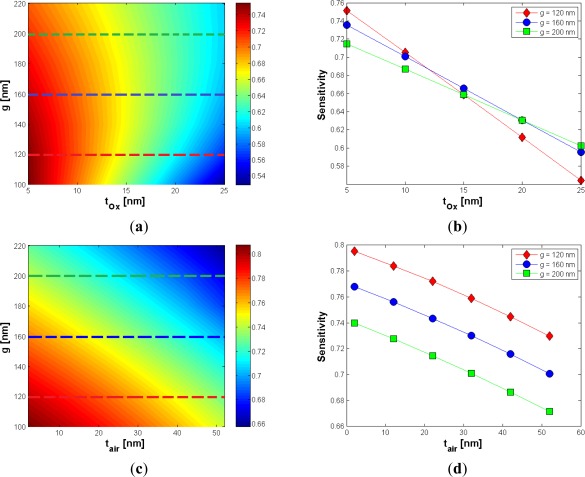
(**a**) Sensitivity *versus g* and *t_ox_*, when *W* = 220 nm and *H* = 220 nm; (**b**) Sensitivity *versus t_ox_* for different values of *g*; (**c**) Sensitivity *versus g* and *t_air_*, when *W* = 220 nm and *H* = 220 nm; (**d**) Sensitivity *versus t_air_* for different values of *g*.

Regarding the additional oxide layer, we have found a linear dependence between sensitivity and *t_ox_*, as in Figure 9(a,b). Our simulations show that the wider the gap, the smaller the absolute value of Ψ_t_ox__, in agreement with considerations for *Γ_c_*. In Figure 9(c,d) the influence of air bubbles is shown.

For oxide layers less than 25 nm thick, a good choice for the gap width is *g* = 160 nm, where *S* > 0.6 can be always achieved. About the influence of air bubble, as in Figure 9(c,d), it can be shown that higher values of *g* produce a slight deterioration of fabrication tolerances (Ψ_t_air__). Such a behavior again agrees with that shown for *Γ_c_*. However, an air bubble has a very low effect, compared to all previously analyzed parameters, according to data reported in Table 2.

In conclusion, a SOI slot waveguide optimized for sensing, simultaneously matching requisites of high sensitivity (*S* > 0.72) and high fabrication tolerances, can be obtained with *H* = 220 nm, *W* = 220 nm and *g* ranging between 120 and 220 nm, depending on the available technology. Table 2 summarizes the main results of our optimization.

### 3.3. Slot Waveguides with High Aspect Ratio

In this Section, an analysis of fabrication tolerances about slot waveguides with high aspect ratio (*i.e*., several hundred nanometers for *H*) is performed, in order to achieve an optimized and robust design for ultra high sensitivity slot waveguide-based photonic sensors. In fact, it could be demonstrated that the higher the silicon wires, the higher the sensitivity of the device.

An important issue for slot waveguide sensors is the single mode behavior, if an interferometer geometry is used. In order to define an analytical criterion to distinguish between TE-like, TM-like and hybrid modes, we have defined the parameters *k_TE_*, *k_TM_* and *χ* as follows:

(8) $$k_{\mathrm{TE}}=\frac{\iint_{\infty }{\left|E_{x}\right|}^{2}\mathrm{dxdy}}{\iint_{\infty }{\left|\vec{E}\right|}^{2}\mathrm{dxdy}};\;k_{\mathrm{TM}}=\frac{\iint_{\infty }{\left|E_{y}\right|}^{2}\mathrm{dxdy}}{\iint_{\infty }{\left|\vec{E}\right|}^{2}\mathrm{dxdy}};\;\chi =\frac{k_{\mathrm{TE}}}{k_{\mathrm{TM}}}$$

The meaning of the symbols should be clear, considering that a TE-like mode has the E_x_ component as the major component, and a TM-like mode has the major component of the electric field oriented in the *y* direction, according with axes orientations shown in Figure 1. In our analysis, a guided mode has been considered TE-like for *χ* ≥ 10, TM-like for *χ* ≤ 0.1 and hybrid for 0.1 < χ < 10. Such a criterion is enough to distinguish TE-like and TM-like modes in almost each circumstance. However, for guided modes with *χ* very close to 0.1 or 10, a rough application of the criterion could lead to a wrong choice. In these cases, a direct evaluation of *k_TE_* and *k_TM_* will eliminate the ambiguity related to χ value.

**Figure 10.**
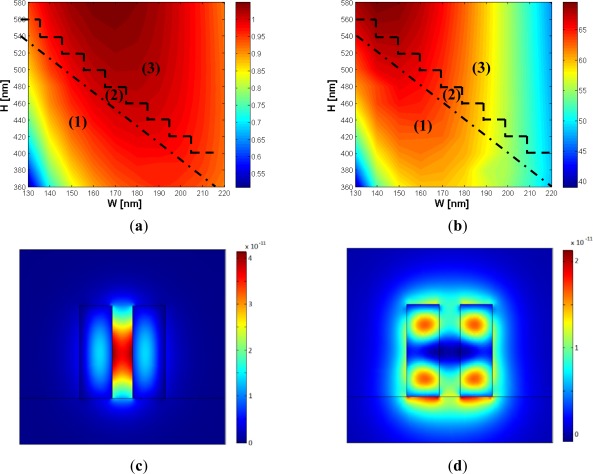
Sensitivity (**a**) and Γ_c_ (**b**) *versus W* and *H* for *g* = 120 nm. In region (1) only the fundamental TE-like mode is guided (single mode area). In region (2) at least one hybrid mode is supported, with *χ* typically ranging between 0.1 and 0.8. Region (3) depends on the assumed step resolution of parameters, so a single mode or multi mode excitation could occur; (**c**) fundamental slot mode field distribution for *W* =190 nm, *H* = 540 nm and *g* = 120 nm (**d**) hybrid mode field distribution for *W* = 190 nm, *H* = 540 nm and *g* = 120 nm.

In the design of a single mode slot waveguide with high aspect ratio, the choice of the gap width becomes critical. In fact, the smaller the gap, the higher the effective index of the waveguide modes, so a single mode behavior becomes extremely difficult to be achieved. On the other hand, sensor performances are significantly deteriorated for large values of *g*, making not convenient the use of high aspect ratio slot waveguides. We have found *g* = 120 nm to be a very good trade-off between single mode behavior, technology limitations and sensing performances. In Figure 10(a,b), a contour map of the sensitivity and Γ_c_ *versus W* and *H* is shown, for *g* = 120 nm.

In region (1) of the contour maps of Figure 10(a,b), only the fundamental TE-like mode has been found to be supported by the structure, with a *χ* coefficient as large as 40 or more. Region (2) supports always one hybrid mode with 0.1 < *χ* < 0.8 (multi mode region), in agreement with the criterion discussed above. It is evident from Figure 10(a,b) that the single mode behavior imposes an upper limit to both sensitivity and power confinement factor achievable by the slot waveguide, since the region of maximum sensitivity can be found in the multi modal area. Due to this reason, when single mode regime is requested, a compromise between performances and fabrication tolerances is required, in particular with respect to the slanted walls angle.

The points on the dashed line are calculated points, so they own to the multimodal region. Region (3) is a region not covered by our simulations, having a resolution *ΔW* = 10 nm and *ΔH* = 20 nm, so the single or multi mode behavior will depend on the specific calculation point.

A choice of *W* and *H* close to the dot-dashed line in Figure 10 could induce multi modal behavior, due to both technologically induced variations of *W* and *H* and small values of *ϑ*. In fact, the sidewall angle becomes a fundamental parameter to be considered in order to guarantee hybrid modes beyond the cut-off condition. A general assumption is that the wider the silicon wires, the smaller the angle *ϑ* for which the hybrid mode starts to be guided, so the optimized design will obviously depend on the available technology.

**Figure 11.**
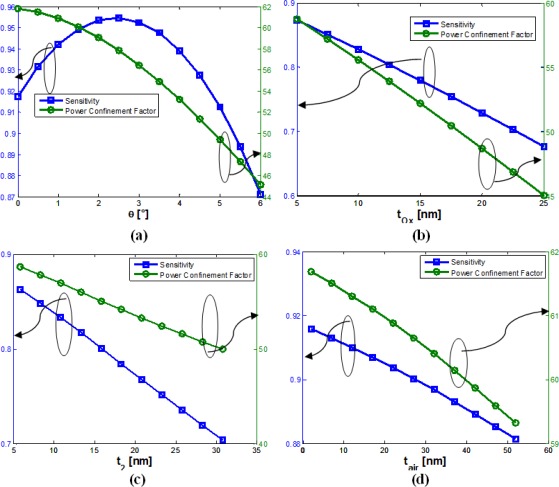
Sensitivity (blue curve) and Γ_c_ (green curve) *versus ϑ* (**a**), *t_ox_* (**b**), *t_2_* (**c**) and *t_air_* (**d**), for *W* = 155 nm, *g* = 120 nm and *H* = 400 nm.

Since *ϑ* = 6° is a practically achievable value for SOI technology [9,17,18], parameters *g* = 120 nm, *W* = 155 nm and *H* = 400 nm are found to be the optimal choice, simultaneously achieving best fabrication tolerances with respect to sensitivity and single mode behavior. In fact, *S* = 0.92 has been calculated, showing a very low dependence with respect to both *H* and *W*. Then, practical advantages cannot be reached with an increase of *W* or *H*, without compromising the single mode behavior. On the other hand, this optimal choice guarantees a large tolerance for the silicon wires width, exhibiting a single mode behavior for *W* < 200 nm. This is a very important issue, due to the fact that *W* is dependent on both photo-lithography and etching processes, differently from the height of the slot waveguide. Figure 11(a–d) show the variation of both sensitivity and power confinement factor with respect to several technological parameters.

Table 3 summarizes the calculated fabrication tolerances in terms of both sensitivity and Γ_c_.

## 4. Coupling into a Slot Waveguide

The problem of coupling light into the slot waveguide is still a critical aspect for slot waveguide sensors [12–14], since the mode profile of a typical rib or strip waveguide has a very different shape with respect to a slot mode profile. Another critical aspect of coupling is the spot size dimension of the fundamental mode, since the slot waveguide forces the field to be confined into a sub-micrometer region. Then, an efficient strip-slot waveguide input and output coupler becomes a fundamental component in order to reduce both device insertion losses and back-reflections, simultaneously optimizing the power coupling into the cladding region for sensing purposes. To this aim, we propose an ultra-compact device, particularly suitable for SOI technology.

**Figure 12.**
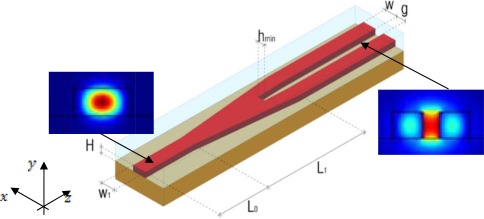
Strip-slot coupler geometry. In the insets, E_x_ field distributions at the starting and ending section of the coupler are shown (calculated by FEM).

Taking advantage from considerations made till now in terms of fabrication tolerances and sensitivity optimization, we have focused our work on low aspect ratio slot waveguides, optimized as in Section 3.2, with *W* = 220 nm, *H* = 220 nm and *g* = 120 nm. The slot coupler geometry is sketched in Figure 12. The device consists of three parts: the first part is a single mode silicon wire waveguide, having width *W_1_* = 510 nm and height *H* = 220 nm (the same as the slot waveguide silicon wires for best technology compatibility). The length *L_0_* of this input waveguide in not a critical parameter, so it would depend only by the specific application (in our simulations *L_0_* = 1 μm). The function of this input waveguide is only to drive the light at the second part of the coupler starting section. This second part consists of a *L_1_* long, tapered Y-branch waveguide.

The input width of both branches is *W_1_/2*, while their output width is *W*. At the output section, the gap between the two silicon wires is *g*. The third part of the coupler is simply the slot waveguide, consisting of two parallel photonic wires of width *W* = 220 nm, separated by a gap *g* = 120 nm. An ultra-high coupling efficiency can be achieved with a short taper length *L_1_* = 4 μm or less, enabling the device to be extremely compact. A remarkable consideration is that the simplicity of this coupling geometry strongly minimizes the technological steps, limiting the occurrence of technologically induced differences with respect to the designed ideal geometry and, then, making the device very robust in terms of fabrication tolerances.

**Figure 13.**
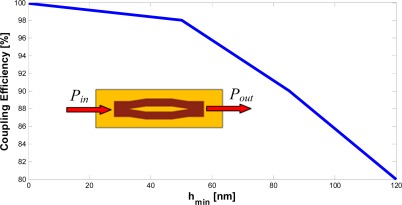
Coupling efficiency *versu*s *h_min_* for the proposed strip-slot-strip coupler (in the inset), as calculated by 3D FDTD.

In order to estimate the coupling efficiency, we have considered a slot waveguide with both an input and output coupler (see the inset in Figure 13). The coupling efficiency has been calculated as [14]:

(9) $$\eta =\frac{P_{\mathrm{out}}}{P_{\mathrm{in}}}$$

where *P_in_* is the power launched in the input waveguide and *P_out_* is the power leaving the output waveguide. Both BPM and FDTD full vectorial 3D simulations [23] have been used to simulate this device, evaluating a coupling efficiency which approaches 100% in both cases. With reference to Figure 12, the influence of a silicon etching residue at the starting section of the Y-branch can be estimated by taking into account the value of *h_min_* in the optimization process. In fact, we have observed that this is the most important parameter affecting the coupling efficiency of the device. It is intuitive that the optimum situation can be achieved with *h_min_* = 0, which cannot be easily obtained in practical fabrication process. However, properly optimizing the taper length and the input waveguide width, it can be possible to reduce the influence of *h_min_* on the coupling efficiency at values less than 2%. We have demonstrated that, for a taper length of 4 μm and *h_min_* ≤ 50 nm, the coupling efficiency never decreases under 98% (see Figure 13). Moreover, the coupling efficiency still remains very high (80%) in case of abrupt transition between strip and slot region (*h_min_* = *g* = 120 nm). In Table 4 the values of cladding power confinement factors are summarized for designed strip-slot coupler, as calculated by 3D FDTD and BPM simulations. and compared with values estimated by a full vectorial 2D FEM analysis.

The FDTD power confinement factors have been calculated at the central z-section of the slot waveguide, while the FEM confinement factors have been calculated with a modal analysis performed on the slot waveguide cross section, regardless of the field shape and effective index at the lower z-sections. We have found a good matching with both FDTD and FEM results, confirming the high coupling efficiency of the proposed device. Thus, the strip-slot-strip coupling efficiency can approach 100%, while the one step strip-slot coupling efficiency is very close to 48%. The high confinement factors in cladding given by Table 4 confirm how this coupler is highly suitable for exciting slot waveguide based photonic sensors designed for monitoring liquid analytes. In our opinion, this simple approach appears to be more practical and efficient than other technological solutions presented in literature [14], usually involving complicated tapering sections.

## 5. Conclusions

In this paper, we have performed an accurate analysis of SOI slot waveguide fabrication tolerances, for both 220 nm thick and several hundred nanometers thick slot waveguides, demonstrating the fundamental role of the gap region dimension to obtain a robust design, simultaneously optimizing both cladding power confinement factor and sensitivity for either absorption or homogeneous sensing devices, respectively. An analysis of single mode behavior for high aspect ratio slot waveguides has been also performed, in order to provide geometrical limits for the design of the waveguide. Finally, a very compact and efficient strip-slot coupler has been investigated. The strip-slot-strip coupling efficiency of the proposed device has been demonstrated to be close to 100% with a very good feature for optical sensing purposes, while the power confinement factor in the cladding region (including slot) approaches the maximum theoretical value (48.4%), as predicted by 2D full-vectorial FEM analysis and confirmed by 3D FDTD. The design of the coupler has been performed by taking into account the technological parameters too, and very good fabrication tolerances have been demonstrated.

## Figures and Tables

**Table 1.** Fabrication tolerances with respect to Γ_c_ for several technological parameters in case of *absorption sensing* schemes.

***g* [nm]**	**Γ_c_ [%]**	**Ψ_ϑ_ [%/°]**	**Ψ_t2_ [%/nm]**	**Ψ_tox_ [%/nm]**	**Ψ_tair_ [%/nm]**
120	49.41	−1.0220	−0.2648	−0.4922	−0.0231
140	49.29	−0.8721	−0.2587	−0.4173	−0.0237
160	49.00	−0.7534	−0.2493	−0.3601	−0.0240
180	48.48	−0.6442	−0.2374	−0.3149	−0.0249
200	47.98	−0.5555	−0.2255	−0.2781	−0.0250
220	47.35	−0.4661	−0.2134	−0.2474	−0.0312

**Table 2.** Fabrication tolerances with respect to *S* for several technological parameters in case of homogeneous sensing schemes.

***g* [nm]**	***S***	**Ψ_ϑ_ [deg^−1^]**	**Ψ_t2_ [nm^−1^]**	**Ψ_tox_ [nm^−1^]**	**Ψ_tair_ [nm^−1^]**
120	0.7984	−0.009203	−0.00612	−0.009356	−0.001310
140	0.7847	−0.006600	−0.00598	−0.008005	−0.001324
160	0.7718	−0.004752	−0.00587	−0.007005	−0.001339
180	0.7574	−0.003084	−0.00573	−0.006230	−0.001348
200	0.7432	−0.001650	−0.00557	−0.005613	−0.001369
220	0.7294	−0.000360	−0.00542	−0.005108	−0.001387

**Table 3.** Fabrication tolerances for *S* (homogeneous sensing) and Γ_c_ (absorption sensing) for all considered technological parameters (Ψ_ϑ_ calculated with *ϑ_max_* = 6°).

		
		**Ψ_ϑ_**	**Ψ_t2_**	**Ψ_tox_**	**Ψ_tair_**
***S***	0.91745	−0.0077 deg^−1^	−0.0064 nm^−1^	−0.0099 nm^−1^	−0.000687 nm^−1^
**Γ_c_**	0.61775	−2.78 %/**°**	−0.35 %/nm	−0.69 %/nm	−0.047 %/nm

**Table 4.** Comparison between 3D FDTD and 2D Full-vectorial FEM calculated power confinement factors, for *h_min_* equal to 0 and 50 nm, respectively.

***h_min_***	**Numerical method**	**Γ_c_**	**Γ_Si_**	**Γ_Ox_**
0	Full-vectorial 2D FEM	48.4%	22.4%	29.2%
	3D FDTD	47.2%	27.7%	25.1%
50 nm	Full-vectorial 2D FEM	48.4%	22.4%	29.2%
	3D FDTD	47.5%	27.7%	24.8%
